# The Influence of Packaging Color on Consumer Perceptions of Healthfulness: A Systematic Review and Theoretical Framework

**DOI:** 10.3390/foods12213911

**Published:** 2023-10-25

**Authors:** Katharina Steiner, Arnd Florack

**Affiliations:** Department of Psychology, University of Vienna, 1010 Vienna, Austria; a00702806@unet.univie.ac.at

**Keywords:** color, packaging, health perception, consumer, design, product evaluation

## Abstract

When consumers evaluate a new product, packaging design plays a critical role. In particular, packaging color is a dominant design cue that influences consumer perception of a product. Several studies have investigated the influence of color on taste. However, there is limited research on the influence of packaging color on consumer health perception. As healthy eating is a focus for many consumers and public decision-makers, more knowledge is needed. The aim of this review is to provide an overview of empirical studies that have investigated the influence of packaging color on consumers’ health perceptions and to provide a psychological explanation for the observed effects. The systematic review includes 20 empirical studies across different product groups. The results show that packaging color influences consumers’ health perceptions. We argue that the influence of packaging color on consumer health perceptions can be explained by the following mechanisms, which are not mutually exclusive: (1) consumers rely on a color as an explicit signal for health; (2) colors are associated with beliefs that indirectly influence health assessments; and (3) colors trigger mental simulations that influence health assessments. In addition, we provide suggestions for further research that will contribute to a better understanding of when and how packaging color can help consumers make healthier food choices.

## 1. Introduction

In today’s fast-paced environment, consumers have an average of only 2.5 s to select a product from a wide range of options on the shelf [1]. Therefore, the first impression of a product, which often comes from the product’s packaging, plays a critical role in product evaluation and purchase decisions. Time constraints prevent consumers from processing detailed product information, and they often rely on visual cues for product evaluation. Several studies have investigated the influence of different visual cues on product evaluation and perception [2,3,4,5]. In particular, packaging color is often used as a marketing tool, and various studies have demonstrated an influence of packaging color on product evaluation, e.g., [5,6,7,8]. Indeed, color is one of the most dominant design cues on product packages [7].

While the influence of packaging color on taste perception has been the subject of many studies (see [9] for a review), research on the influence of packaging color on health perception is scarce. Accordingly, reviews on this topic are limited [10]. Given the high rates of obesity among adults and children in developed countries and obesity as a high-risk factor for noncommunicable diseases and disability [11], it is necessary to better understand the factors that influence consumer perceptions of health. Understanding how consumers use and understand color cues to form perceptions of a product’s healthiness can help marketers of healthy product alternatives successfully design their products and local governments identify interventions.

The aim of this paper is to provide a summary of the current state of the literature on how packaging color influences consumers’ perceptions of healthiness. Because there is a lack of coherent theory explaining the effects of packaging color on health perception, we discuss psychological concepts that may explain the influence of packaging color on consumers’ health perceptions and subsequently build a theoretical framework. Also, we will identify variables that moderate the influence of packaging color on consumer health perceptions and offer research suggestions based on the results of this review.

## 2. Method

To conduct an objective review of the current state of the literature, we used the Preferred Reporting Items for Systematic Reviews and Meta-Analysis (PRISMA) guidelines [12].

### 2.1. Study Eligibility Criteria

This review included studies that investigated the effect of packaging color on consumer health perceptions of packaged foods. Specific inclusion criteria were as follows: (a) manipulation of packaging color while holding the other factors constant in at least one other condition; (b) healthiness rating as the dependent variable; (c) investigation of the effect for packaged foods, excluding studies on the design of restaurant menus, dietary supplements, and addictive substances such as cigarettes and drugs; (d) observation of the effect for adults, not children. Studies that did not systematically vary color or color variations as a single cue were excluded because these studies would not allow inferences about the effect of color. In addition, studies that examined the effect of transparent versus non-transparent packaging were excluded, as seeing a product goes beyond the effects of varying the color of a product’s packaging.

### 2.2. Search Strategy

A systematic literature search was conducted in the abstract and citation database of peer-reviewed literature, Scopus, which covers worldwide research in science, technology, medicine, the social sciences, and the arts and humanities from 1970 to the present. The objective was to find research that investigated the influence of packaging color on consumer health perception. Therefore, the following keyword combinations were used: ‘health’ OR ‘healthiness’ OR ‘healthy’ OR ‘healthfulness’ AND ‘color’ AND ‘package’ OR ‘packaging’ and ‘food’ and ‘consumer’. The restriction was English language.

### 2.3. Study Selection and Results

**Figure 1:**
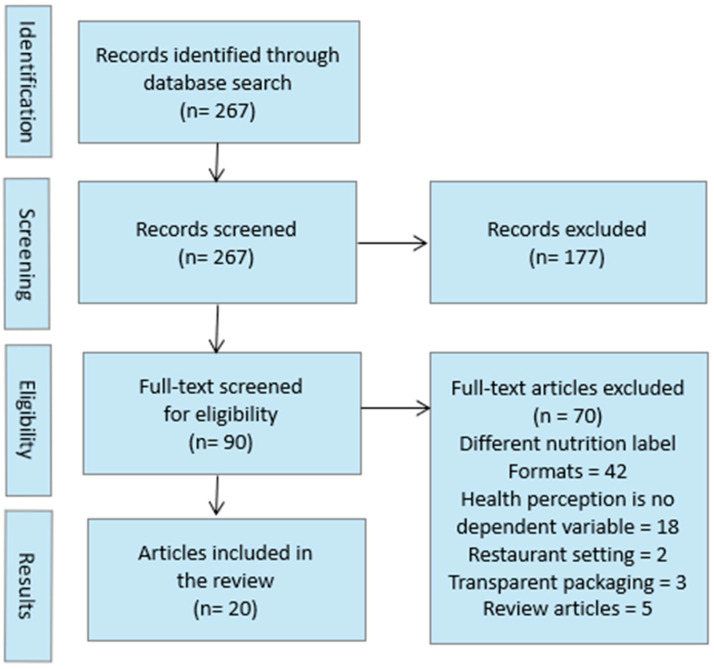
PRISMA (Preferred Reporting Items for Systematic Reviews and Meta-Analysis) chart of search strategy.

A search of Scopus using the keywords described above returned 267 results. The relevance of these results was assessed in two steps. First, all titles and abstracts were screened, and clearly irrelevant studies were removed. Second, the full text of the remaining 90 studies was evaluated, and studies that did not meet the eligibility criteria were excluded. The final sample consisted of 20 articles with *K* = 35 individual studies that were included in the final qualitative synthesis (Figure 1). To provide an overview of the results and make them more readily understandable, sample characteristics, research design, and main findings are presented in Table 1.

## 3. Results

To describe the results of the selected studies, we grouped them according to the different color stimuli they manipulated to observe the influence of packaging color on consumer perceptions of healthfulness.

The studies reviewed used different methods, such as focus groups and questionnaires, to measure the influence of packaging color on consumers’ health perceptions, with congruent results. However, the studies did not apply physiological measures.

### 3.1. Warm vs. Cool Color Hues

One strand of literature analyzed the influence of long-wavelength, or warm, color hues such as red and yellow vs. short-wavelength, or cool, color hues such as blue and green on consumers’ health perceptions of products. The differentiation between warm and cold colors is not new in advertising. Researchers have studied the impact of warm and cool colors in several settings [13]. A basic idea in this research is that consumers respond with a positive feeling of calmness to cool colors, whereas warm colors are more likely to be associated with arousal and attention. Correspondingly, cool packaging colors have been associated with higher health perceptions than warm packaging colors in most but not all studies examining health perceptions [6,14,15,16,17]. Below, we describe the studies comparing the effects of warm vs. cool colors on health perceptions in detail. 

The study of Hallez et al. [16] investigated how packaging design with warm vs. cool colors and the presence vs. absence of nutritional and ecological claims influenced consumer evaluations. They demonstrated that green and blue packaging designs, representing cool colors, led to higher health and sustainability perceptions [16]. These findings are consistent with the study of Plasek et al. [14], who observed that the color combination white-blue is perceived as healthier than white-red and white-green, but white-green is perceived as healthier than white-red. In addition to color, this study also observed the influence of shape, the presence or absence of health claims, claims related to product ingredients, and country of origin on consumer health perceptions. Of these extrinsic product cues examined, color had the strongest influence on consumer health perceptions [14]. 

Karnal et al. [4] analyzed the influence of soft drink packaging color (yellow vs. red) on consumers’ health perceptions. Participants perceived products with yellow-colored packaging as healthier than those with red-colored packaging. They also perceived yellow as less heavy, less warm, and less arousing than red. However, the direct effects of color on heaviness and health remained even after controlling for arousal and warmth [4].

Sant’Anna et al. [17] studied the effects of package color on health perceptions and consumer choices. They found that consumers associated blue packaging with healthier products when shopping for products with reduced sodium. In their study, the blue packaging color compared to red, combined with the phrase indicating the percentage of sodium reduction, increased the likelihood that participants chose a cracker with reduced sodium when searching for such products [17].

In contrast to the described studies that found a positive influence of green and blue versus red packaging colors on consumers’ perceptions of healthiness, in Marque de Rosa et al.’s [18] study, participants rated cookies in red-to-yellow packaging as healthier than those in blue-to-green packaging. Another study found no effect of warm vs. cold color on healthiness. In a study by Sucapane et al. [19], adding a green color to a “plant-based” product and a red color to a “meat alternative” did not increase the differences in healthiness perceptions between the two product options.

There are some indications that the effect of warm and cool colors may vary depending on the food category. Based on a qualitative study, Wąsowicz et al. [20] concluded that, depending on the food category, consumers can associate the cold colors blue and green but also the warm colors yellow and red with healthiness. For example, in the frozen pizza category, participants associated the color red with healthiness. According to the researchers, this was presumably because it was perceived to indicate the presence of fruits and vegetables. However, in the yogurt category, red was associated with artificial coloring and regarded as a signal of low healthiness [20].

Huang and Lu [6] studied the hedonic quality of products as another moderator of the influence of warm and blue packaging colors on consumers’ health perceptions. In some product categories, such as ice cream or potato chips, products primarily serve hedonic goals such as affective pleasure, while in others, such as milk, they serve functional goals such as satisfying physical needs (e.g., thirst). For products that serve utilitarian goals, the researchers found that blue packaged products were perceived as healthier than red packaged products. For products that serve hedonic goals, however, they did not observe an influence of packaging color on the healthiness assessments of the products [6]. 

### 3.2. Color Brightness and Saturation

Another stream of literature investigated the influence of other aspects of color beyond hue, such as brightness and saturation. Researchers found that consumers perceive product packages with higher brightness and lower saturation as healthier, e.g., [21,22]. 

Mai et al. [21] found an association between light-colored packaging and health perceptions. In their study, subjects had to rate product packaging from different food categories, including healthy and unhealthy categories, that differed in color lightness. All other packaging parameters, such as hue, saturation, and package size, were held constant. Light-colored packaging led to higher healthiness perceptions but lower tastiness perceptions and purchase intention. Mead and Richerson [22] were able to replicate these findings, showing across four experiments in different food categories (healthy and unhealthy) and across a variety of hues that consumers associate highly saturated packaging colors with unhealthy foods. 

In contrast to these findings, Kunz et al. [5] (Study 2) found a positive relationship between packaging color saturation and perceived product healthiness. In this study, the researchers experimentally varied the saturation of 20 images of non-alcoholic beverages (e.g., juices and smoothies) by increasing or decreasing the saturation of the original packaging by 25 percent. They found that participants perceived the products as fresher and healthier when the packages were more saturated than when they were less saturated.

### 3.3. Variations of More Than One Aspect of Color

Other authors compared experimental conditions varying in more than one specific aspect of color. Tijssen et al. [23], for example, compared packages of a diary drink and a sausage with varying color hues (blue, green, red, purple), brightness (high vs. low), and saturation (high vs. low) of the packaging color. All other attributes of the packaging were kept constant. This resulted in 12 different tested packaging images for each product. The researchers observed that packaging with less vibrant, cooler colors (e.g., blue), higher brightness, and lower saturation was implicitly associated with healthiness and explicitly perceived as healthier but less attractive than product packaging with warmer colors (red), lower brightness, and higher saturation. 

In one study, Schnurr [24] (Study 1) varied the packaging of nuts as a snack and measured health and taste perceptions and expected enjoyment of the product. In one condition, the packaging was presented with bubbles of different colors (e.g., green, yellow, pink, and blue). In a control condition, there were no bubbles on the package. Participants rated the product as less healthy and tastier in the colored bubbles condition. In a mediation analysis, these effects were mediated by the expected enjoyment of eating the product, with more enjoyment expected when the colored package was presented than when the plain package was presented. In another study [24] (Study 3), the researchers presented participants with a cereal package and compared a control condition with a plain package to a condition in which bubbles of different shades of pink were printed on the package. They also evaluated whether the consumption motive played a moderating role and presented the packages with text related to enjoyment (e.g., unique pleasure) or health (e.g., reduced calories). The color variation only had an effect on health ratings when the text focused on health. In this condition, participants rated the healthiness of the product lower when the colored bubbles were on the product packaging compared to the plain packaging.

### 3.4. Color Coding of Front-of-Pack Nutrition Labels

Another line of research has examined the effect of the color of the front-of-pack (FOP) nutrition label, such as the calorie label, on consumer perceptions of healthiness. In this line of research, studies have often but not exclusively compared the effects of the warm color red and the cool color green. In addition, a few studies have used different variations of a color or control groups with black and white labels. We present this research as a separate category because it is clear that the goal of using these colors is to support understanding of the calorie content label. A common finding is that the use of green- (or red-) colored FOP nutrition labels helped consumers identify a healthy product [25,26,27,28]. 

Aschemann-Witzel et al. [25] found that German consumers who were instructed to select healthy products made more healthy choices when the nutrition label was color-coded according to a traffic light system (red = not healthy, amber = moderately healthy, green = healthy) compared to monochromatic nutrition labels (different levels of blue saturation with lower saturation associated with the healthier product), while holding nutritional values constant. Nutrition labels with monochromatic variations led to healthier food choices compared to uncolored nutrition labels. However, color variations only had an impact on the choice of healthier products in Germany, not in Poland, and only when participants were asked to choose a healthy product [25].

Pettigrew et al. [26] presented participants with choices of three breakfast cereals and asked them to choose the healthiest product. They found that traffic-light-colored versus black and white nutrition labels facilitated the correct choice. Interestingly, the researchers found this effect of traffic-light-colored labels in almost all countries observed. In Australia, China, India, New Zealand, the United Kingdom, and the United States, colored labels outperformed uncolored labels in correctly choosing the healthiest product. Only in Canada did participants understand the health information better with monochrome labels than with colored labels. 

While Aschemann-Witzel et al. [25] and Pettigrew et al. [26] studied the classic traffic light nutrition label, Nyilasy et al. [29] investigated with US consumers whether coloring individual nutrients could confuse consumers because healthy products often contain healthy and unhealthy nutrients. The authors hypothesized that in such a situation, a color scheme might draw undue attention to unhealthy nutrients and less attention to healthy nutrients marked in green. In the study, the researchers presented participants with product images for bread, rice, cereal, and potato chips. In one condition, they presented the product packaging with healthy nutrients (e.g., protein and vitamins) colored green and unhealthy nutrients (e.g., fat and sugar) colored red. In the second condition, they reversed the colors. Finally, in a third condition, they used black and white labels. In line with their expectations, the researchers found that participants showed the strongest differentiation between healthy and unhealthy products when a black and white color scheme was used. This effect was only found for healthy foods, but not for unhealthy foods.

While the studies reported above examined whether colored labels aided in the understanding of nutritional information, Schuldt [27] investigated whether colored labels could directly affect health perceptions. The researcher compared conditions in which the color of a chocolate candy bar label varied while the calorie content remained the same and found that consumers rated the chocolate candy bar as healthier when presented with a green vs. red calorie label. When comparing a green calorie label to a white calorie label, only individuals who generally focused on healthy eating perceived the candy bar with the green calorie label as healthier. However, these findings could not be replicated in Vasiljevic et al.’s [30] study, which also held calorie content constant. In this study, the researchers found that participants rated cereal and chocolate bars with a white label and a smiling emoticon as healthier than those with green or red labels. 

In another study, Cabrera et al. [15] varied the color and shape of a warning label and examined the effect on health perceptions and attention. The researchers used three different conditions of shape (triangle, triangle with vertex, and octagon) and two conditions of color (red and black). Overall, color had a greater effect on health perception than the variation in shape [15]. The researchers observed that participants perceived products with a black label as less healthy than products with a red label. Furthermore, they found that the combination of a red FOP warning label with an octagonal shape, which is often used in traffic signals to communicate risk or danger, was perceived as the least healthy combination compared to other color and shape combinations. 

Meng and Chan [31] investigated whether gender moderates the influence of colored nutrition labels on perceptions of product healthiness. They held the nutritional information constant across color conditions and found that men were more responsive to color, perceiving crackers with a green-colored nutrition label as healthier and crackers with a red-colored nutrition label as less healthy compared to a black-colored label. For women, label color had no effect on their perceptions of product healthiness [31].

**Table 1:** List of studies exploring the influence of packaging color on consumers healthiness perception and their key findings.

Reference	Analytical Method	Product	Country	Key Findings
Aschemann-Witzel et al. [25]	choice task and questionnaire	20 sweet and 20 salty snacks	Germany and Poland	When German consumers were explicitly instructed to make healthy food choices, color-coded nutrition labels led to healthier food choices compared to uncolored nutrition labels. This effect was stronger when the nutrition label was color-coded according to a traffic light system (green = healthy, orange = moderately healthy, red = unhealthy) than when it was monochromatic (different shades of blue). This effect was only observed in Germany and not in Poland. The presence or format of a nutrition label had no effect on the healthiness of consumer choices when consumers could choose according to their preferences.
Hallez et al. [16]	choice task and questionnaire	soft drinks, salty snacks	Belgium	Cool packaging colors, such as green and blue, compared to warm packaging colors, such as red and orange, led to higher perceptions of health and sustainability for drinks and foods. Cool packaged drinks were perceived as less tasty and were less likely to be selected compared to warm packaged drinks. This effect was not observed in the snacks category.
Huang and Lu [6]	questionnaire	milk, cereal, yogurt, potato chips, ice cream, ice tea	Canada	Utilitarian food products, which are consumed mainly to satisfy physical needs such as thirst or hunger, were perceived as healthier when the packaging color was blue compared to red. For hedonic food products, which are consumed mainly for affective pleasure, no effect of packaging color on healthiness perception was observed. Higher perceived product healthiness also led to higher purchase intention.
Kunz et al. [5]	questionnaire	snacks and drinks	Austria	A positive correlation between healthiness and tastiness was observed. Presenting the images only in grayscale vs. color had no effect on healthiness ratings. Moreover, increased versus decreased color saturation led to higher healthiness and tastiness ratings, and this effect was mediated by the perceived freshness of the products.
Mai et al. [21]	questionnaire and Implicit Association Test	pizza, chocolate, yoghurt, cream cheese, potato chips, fruit bar, juice	Germany	Light-colored product packaging led to a higher perception of healthiness, and this effect was observed even after consumers had tasted the product. However, light packaging color had a negative impact on taste, leading to lower purchase intention, especially when consumers had to make heuristic taste inferences and when health was not the primary consumption goal.
Marques da Rosa et al. [18]	questionnaire	buttery and cereal cookies	Brazil	Buttery and cereal cookies were perceived as healthier in a red-to-yellow package than in a blue-to-green package. In addition, the healthiness of cookies in angular packaging was rated higher than that of cookies in round packaging. No interaction effects were detected.
Mead and Richerson [22]	questionnaire	potato chips and nutritional bars	USA	Consumers associate vivid, highly color-saturated food packages with less healthy foods compared to less color-saturated food packages. This effect is mediated by conceptual fluency, as consumers are regularly exposed to unhealthy foods in highly saturated packaging and have learned this association. Consumers with higher levels of nutritional knowledge are less likely to judge foods based on packaging color saturation. On the contrary, consumers with high vs. low restrained eating behaviors are more likely to make inferences about the healthiness of a product based on packaging color saturation.
Meng and Chan [31]	questionnaire	crackers	USA	A positive influence of a green nutrition label and a negative influence of a red nutrition label compared to a black nutrition label on the healthiness perception of a product was observed for men but not for women. Text-based health information on the nutrition label influenced health perceptions for both genders.
Nyilasy et al. [29]	questionnaire	cereals	USA	Consumers perceived healthy cereals with red and green nutrition labels as less healthy than those with uncolored nutrition labels, regardless of which nutrient values were colored green and red. For unhealthy foods, there was no effect of colored versus uncolored nutrition labels on consumers’ perceptions of healthiness.
Pettigrew et al. [26]	choice task and questionnaire	cereals	Australia, China, India, New Zealand, UK, USA	In Australia, China, India, New Zealand, the United Kingdom, and the United States, consumers understood traffic-light-colored nutrition labels (green = healthy, orange = moderately healthy, red = unhealthy) better than uncolored labels. Only in Canada did participants understand the health ratings better with monochrome than with colored labels. Colored nutrient summary labels were the most understandable compared to monochrome labels with nutrient-specific information.
Pettigrew et al. [28]	choice task and questionnaire	cereals	Australia	Colored nutrition labels were better understood than black and white nutrition labels. The most effective labels included only the color coding and abbreviated star rating, not the detailed nutrient icons.
Plasek [14]	questionnaire	functional smoothie	Hungary	Products with white-blue packaging were four times more likely to be perceived as healthy, and products with white-green packaging were twice as likely to be perceived as healthy as products with white-red packaging. Packaging color had the greatest impact on consumers’ health perceptions compared to product claims related to ingredients, organic origin, health claims, shape, and country of origin.
Sant’anna et al. [17]	focus groups and choice experiment	sodium reduced cracker	Brazil	When consumers were asked to choose a reduced-sodium product, blue versus red packaging combined with the phrase indicating the percentage of sodium reduction increased the likelihood that a reduced-sodium product would be chosen.
Schnurr [24]	questionnaire	nuts and muesli	USA and Europe	A cute, colorful packaging design compared to a less colorful, neutral packaging design leads to a lower perception of healthiness for virtue products that are consumed primarily to satisfy physical needs. This effect was not observed for vice products, which are consumed for their hedonic value. A colorful, cute packaging design creates images of fun in consumers’ minds that they imagine they would experience while consuming the product.
Schuldt [27]	questionnaire	chocolate candy bar	USA	Consumers perceived a candy bar with a green calorie label as healthier than a candy bar with a red calorie label, even though the calorie content presented was the same. When comparing a green to a white calorie label, only consumers who focused on healthy eating perceived the candy bar with the green label as healthier.
Sucapane et al. [19]	questionnaire	plant-based meat alternative products	Canada and USA	A “meat alternative” product descriptor combined with a mismatching green versus matching red packaging color led to lower perceptions of eco-friendliness and trial likelihood. A “plant-based” product descriptor combined with matching green versus mismatching red packaging had a negative effect on predicted satiety. No effect of packaging color on health perceptions was observed.
Tijssen et al. [23]	Implicit Association Test and questionnaire	low-sugar dairy drink and low-fat sausage	Netherlands	The hue, brightness, and saturation of a product’s packaging color influence consumers’ perceptions of its healthiness. Consumers implicitly and explicitly perceive products with cooler colors (e.g., blue), higher brightness, and lower color saturation packaging as healthier but less attractive than products with warmer colors, lower brightness, and higher color saturation.
Vasiljevic et al. [30]	questionnaire	chocolate and cereal bar	UK	Consumers perceived a white label with a smiling emoticon as healthier than a red or green label with a smiling emoticon. The influence of the colored label on consumers’ perception of health was only observed in combination with a smiling emoticon, not with a frowning emoticon or no emoticon.
Vila-López and Küster-Boluda [32]	questionnaire	juice with milk and fruit and candy bar	Spain	Product packaging with blue versus red or black nutrition claims was perceived as healthier. Visual cues (font color) had a stronger influence on consumers’ attitudes towards the product than informative cues (nutrition claim). No differences were found between hedonic and functional products.
Wąsowicz et al. [20]	focus group and questionnaire	pizza, yoghurt	Poland	Certain colors have greater potential to be associated with product healthiness, but this varies across product categories. Consumers perceived blue and green, as well as red and yellow, as healthy, depending on the product category. For example, the color red was perceived as healthy in the pizza category because it indicates the presence of fruits and vegetables, but unhealthy in the yogurt category because red is associated with artificial colors.

## 4. A Cognitive Ecological Color Theory

According to the research outlined above, different packaging colors influence consumers’ perceptions of a product’s healthfulness and help them choose and purchase healthy foods. The findings also show that the effects of color vary across product categories, countries, and individuals. However, there is currently no comprehensive theory to explain when the effects of packaging color on health perceptions should be expected. Therefore, we discuss a cognitive ecological color theory as a starting point for creating a more comprehensive theoretical framework to derive hypotheses about how packaging color affects perceptions of food health and stimulate future research.

The proposed approach is a cognitive one, as it makes statements about intrapsychic processes that are triggered by the perception of color. However, these intrapsychic processes are not considered independent of the environment, cultural conditions, or product categories. Rather, in line with cognitive ecological theories, e.g., [33,34], it is assumed that certain effects of colors on the expectation that a product is healthy can be better explained by taking into account how intrapsychic processes depend on various features of the environment (the ecology), such as how often or rarely a color occurs in a product category. The proposed approach is based on the assumption that cognitive processes have an adaptive function that allows them to adjust to the demands of a given environment [35]. A comparable approach is the so-called color-in-context theory, which is concerned with the effects of color on psychological functioning [36]. For example, it is well known that in a competitive context, the color red can be perceived as a signal of danger, such as when an opponent is angry (e.g., having a red face) [37]. However, the same color can also emphasize sexual attractiveness and thus be associated with a tendency to approach (e.g., a woman using red lipstick) [38]. 

The proposed cognitive ecological theory of color is based on the view that the perception of color has proven to be useful for survival throughout evolution. The perception of color is therefore not completely arbitrary, but humans perceive mainly those colors that have emerged to be functional [39]. For example, the color red has a very high biological significance in many respects. Red indicates certain dangers, such as blood flowing from a wound. But it is also associated with the ripeness of a fruit. So, we assume that there are certain biological predispositions that make it easier for people to learn associations between colors and attributes and also response patterns. However, we also hypothesize that there is an adaptive mechanism for learning meaning from colors that relies on acquiring knowledge and learning associations. 

Based on extant research in marketing and psychology [9,40,41], we hypothesize that the color of labels or product packaging has a meaning that goes beyond the aesthetic embellishment of a product. Learning the specific meanings and associations should occur through psychological learning mechanisms such as declarative and associative learning [42,43]. First, consumers should learn meanings of color as explicit knowledge through communication and observation [44]. For example, the meaning of a colored label could be transmitted by a health campaign. Also, consumers can transfer their knowledge from another domain to the health domain, such as when they use their knowledge about traffic lights to understand a traffic light label system for food products. Second, the implicit meanings of color should result from associative learning [45]. This learning should be subject to the principles of contiguity (association with attributes that occur in close spatial or temporal proximity to the color), frequency (the more frequently the color occurs with the attributes, the stronger the associations), and distinctiveness (when the color occurs more frequently with the attribute than with other colors) [46]. It is important to note that declarative and associative learning are context-sensitive [47]. For example, people quickly learn the explicit knowledge that a colored shirt is appropriate for a vacation, but not for a funeral.

**Figure 2:**
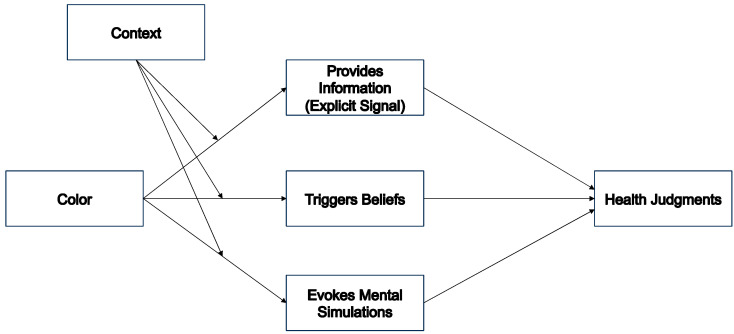
Model showing three pathways of color effects on perceptions of healthiness.

Building on the cognitive ecological framework, we postulate three ways in which colors on packaging or labels can influence expectations about the healthiness of a product (Figure 2). First, we hypothesize that consumers often consciously and deliberately use and seek out colors as informational cues for healthiness—for example, when the color green on a label represents a healthier food (color as information). Second, we suggest that colors may indirectly influence health evaluations through beliefs (colors as triggers of beliefs). We suppose that colors can evoke an impression that leads to the application of a health-related belief. This would be the case if certain color combinations signal fun. Consumers might associate the food with enjoyment and then apply the belief that food cannot provide enjoyment and health at the same time, and therefore the product is more likely to be unhealthy. Third, we postulate that, in contrast to these explicit effects of color, there may also be implicit effects that influence health evaluations (colors influencing mental simulations). These effects are not necessarily conscious and may influence an assessment through a feeling or a halo effect.

### 4.1. The Use of Color as Information

People automatically perceive colors, and the associations people have with colors are automatically activated [48]. It is not possible to look at a product and consciously inhibit the perception of the color. However, the automatic perception of color does not mean that all color influences are unconscious. In fact, a lot of studies suggest that consumers deliberately employ the learned meanings of colors—for instance, when they need to choose the healthiest alternative among several products [28]. A good example for the explicit use of colors as information is the reliance on a warning signal [15]. While the process of relying on these signals can be automated, in critical or ambiguous situations, consumers may explicitly search for such signals, much like a driver looking for a stop sign at a confusing intersection. We summarize these kinds of usage of color as the use of color as information. Whether consumers use the color as information in their health judgments can be a deliberate decision, the application of a simple heuristic, or a habitual response; it reflects that consumers have learned the meaning of the color as declarative knowledge and know the meaning when they are asked for it. 

We assume that consumers learn color meanings when colors co-occur with health perceptions or health information, but that these meanings are also transported through social learning and communication. Therefore, our approach is compatible with the idea that color meanings represent shared knowledge that may vary across cultures [15,20,49]. It is important to note that our approach proposes that consumers can learn context-specific color meanings and is congruent to findings that color meaning differs between product categories. An illustration of context-specific meanings can be found in the study by Wąsowicz et al. [20], where participants stated that red in the frozen pizza category was perceived as healthy because it represented the vegetables contained in a pizza, whereas red in the yogurt category was perceived as unhealthy because it represented artificial coloring. Furthermore, a red-to-yellow packaging color was perceived as healthy for the cookies category in a study by Marque de Rosa et al. [18], while other studies found a negative impact of a red packaging color on consumers’ healthiness perceptions of soft drinks and salty snacks [4,16]. 

The research reviewed above illustrates that color is an important signal of product health in many contexts. However, it is clear that it is only one piece of information among others that can influence consumers’ health judgments, especially when they are making a quick decision. In fact, color is an easily identifiable cue, and the meaning associated with it is immediately apparent when learned as declarative knowledge. This can be seen in Pettigrew’s study [26], where colored traffic light nutrition labels are better understood than monochrome nutrition labels. 

The use of color as information, however, does not only mean that consumers distill healthiness from the color, but that they may begin to process information in more detail when colors do not fit or occur in an unexpected context. We assume that the appearance of a color leads to increased processing and search for a reason for this appearance if it deviates from the learned knowledge. In the study conducted by Nyilasy et al. [29], the authors observed that red labeling of unhealthy nutrients had a more pronounced effect on the evaluation of products perceived as healthy compared to those perceived as unhealthy. Although products that are generally perceived as healthy may contain some unhealthy nutrients (albeit in lower amounts than in unhealthy products), consumers may find red labels on such products unexpected. Nyilasy et al. [29] assume that as a result, consumers are likely to pay more attention and process this information more intensively when it appears on healthy products than when the same information is highlighted in red on a product typically considered unhealthy.

### 4.2. Colors as Triggers of Beliefs

A further influence path of colors on healthiness judgments refers to beliefs that are triggered by colors or color combinations. An often-cited lay belief that influences health perceptions is the unhealthy = tasty belief. Such beliefs can either be learned via experience [34] or through the media and culture [50], or they may be applications of higher-order beliefs. For example, Raghunathan et al. [51] argued that consumers may think that healthy eating is incompatible with eating for pleasure because goals that are considered morally good are usually achieved through effort, not pleasure. Thus, we suggest that cues that communicate enjoyment trigger the unhealthy = tasty belief and lead to lower health judgments in individuals who hold this belief. An example of this is the aforementioned research by Schnurr [24], who found that colorful packaging leads to higher perceptions of taste and lower perceptions of healthiness, because colorfulness is associated with fun and enjoyment.

Another possible lay belief that has not yet received much attention in research on color–health associations is that more expensive foods are expected to be healthier [52]. Thus, packaging colors that signal luxury or low-budget categories, such as black and silver (luxury) or pink (low-budget) in some supermarkets, may influence not only quality but also health expectations if consumers believe that more expensive products are healthier. Hence, colors may trigger beliefs in purchase or consumption contexts that consumers otherwise would not have applied.

### 4.3. Colors Influencing Mental Simulations

The use of color as information or application of a belief does not sufficiently explain how color can incidentally influence healthiness judgments. We argue that in addition to conveying explicit information or triggering a belief, color can also influence consumers’ perceptions of a product’s healthfulness by eliciting mental simulations and the resulting feelings or impulses [5,6,24,27,29].

We suppose that consumers simulate the eating experience when making product choices [53]. When consumers consume unprocessed foods such as fruit, the saturation of the color represents its freshness and should lead to increased taste and health perceptions because rotten food is less healthy than fresh food. Kunz et al. [5] found such an effect for packaged beverages such as juices and smoothies. In their study, packaging with saturated colors led to increased taste and health perceptions compared to packaging with reduced color saturation. We assume that the participants in this study simulated drinking the juices and that the simulation influenced the health and taste judgments. Interestingly, the study also showed that taste and health judgments are congruent in some contexts, as taste and health judgments are sometimes influenced by the same cues such as color saturation [54]. More recent research further underscores the notion that individuals with different learning histories (e.g., from different social classes) may rely on these cues differently, which may help explain why some individuals eat less healthfully than others [55].

Another reason for the importance of the mental simulation path of color effects is that the quality of affective experiences elicited by exposure to a color and the consequences of healthy/unhealthy eating are sometimes similar. For example, the colors green and blue elicit feelings of safety, calm, and relaxation [56]. The same feelings are associated with the consumption of healthy products. According to the proposed mental simulation approach, these evoked emotions can explain the healthier product perception of blue- and green-colored products [6,16,27,32]. On the contrary, warmer colors such as red and orange may trigger feelings of excitement and physical arousal, which are incongruent with feelings associated with healthiness. Hence, the mental simulation account would predict that taste expectations are positively affected and health judgments are negatively affected by these colors, which is in line with the findings reviewed above [6,32,56].

In addition, colors have been shown to influence weight perceptions [4,57]. Following the mental simulation pathway in our model, light colors, such as light blue, could create a feeling of lightness which spills over to healthier perceptions of a product. The aforementioned study of Mai et al. [21] supports this notion. Perceived weight is also affected by different color hues, as red, for example, may make a product or its consumption seem “heavier” than yellow, which may also contribute to the lower perceived healthiness of red-colored packaged foods [4].

Finally, it is important to note that a single color can potentially influence perceptions of health through multiple pathways. Often these pathways are congruent, as illustrated by the influence of red in the previous example. However, opposing effects are also conceivable. It should be possible for a color to be perceived as a direct indicator of healthiness while at the same time inhibiting the mental simulation related to healthy eating experiences that typically correlate with variety, pleasurable sensations, and not feeling too full. If the effects on the different pathways are contradictory, the overall effect of the color would be expected to be diminished. 

## 5. Discussion and Conclusions

The main objectives of this systematic literature review were to summarize the current literature on the influence of packaging color on consumer health perception of a product and to provide a framework to integrate the results and inspire future research.

Overall, the described research shows the different influences of packaging color on consumers’ health perceptions of a product. To better understand the influence of packaging color on health perceptions, we introduced cognitive ecological color theory. Similar theories have been developed in other fields [36]. However, we specified this theory to explain the context-specific color effects on healthiness perceptions and expanded it to three different paths of influence: the use of color as information, the beliefs triggered by colors, and the mental simulations influenced by color. In principle, this theoretical approach can be applied to food color effects in general, particularly to color effects on taste. However, it is important to note that healthiness judgments are different from taste because declarative knowledge plays a greater role in these judgements and because the effects of mental simulation are less obvious for health judgments. 

The evidence that color can influence health judgments is strong. The most obvious evidence is that people use color as an explicit cue to make a judgement. They have declarative knowledge about the meaning of color cues and they apply this knowledge. However, our review illustrated that this pathway of color influence is strongly context-dependent; it varies between product categories, between individuals, and between cultures. It is unlikely that a single color has a strong effect on health judgments across different contexts and for all kinds of food products. If it is at all possible, green could be a candidate for a color that positively influences health judgments across contexts. But if green is used in a product category in which health is unexpected (e.g., chips), its effect might disappear. Hence, future research is needed on much more nuanced aspects of the impact of color on healthiness judgments. In our review, we mentioned a study by Kunz et al. [5] showing that participants rated drinks as healthier when the saturation of the color on the image was higher. Other research studies found that higher color saturation can also be linked to lower healthiness [22,23]. Taking our path of influences into account, it is important to have a closer look at these effects. The question is which path of influence was active in the respective studies. For example, when a consumer perceives a package of a smoothie with the package color representing the content, simulation of consumption might be important and lead to the impression of low freshness when the saturation is low. When a consumer is exposed to a package of chips with the same color, which does not represent the content, simulation makes less sense. It is more likely that the consumer uses a saturated color as a signal for fun and enjoyment. In this case, a consumer might apply the unhealthy = tasty belief and judge the product as less healthy when the package color is saturated. This information is highly relevant for businesses and marketers to successfully design their product packages. As previous studies have shown, healthy product design can have a negative impact on the success of a product [23]. A better understanding of the underlying mechanisms and pathways used can help overcome this barrier and convince consumers to make healthier food choices. 

There are several ways to assess the effects of color through the three different pathways. First, researchers can use self-report measures to assess whether color is explicitly used as information in the health evaluation situation. Second, researchers can measure beliefs that are hypothesized to be elicited by color and health judgments and conduct a mediation analysis to test whether the beliefs mediate the color effects. However, such measures of beliefs may draw participants’ attention to the beliefs, which could strengthen or even dilute the relevance of the beliefs when participants control for the influence [58,59]. Therefore, researchers could use a two-step approach, measuring the relevant beliefs at Time 1 and using an experimental design at Time 2 in which they vary the colors and measure the effects on healthiness perceptions. In this design, individual differences in the beliefs measured at Time 1 should moderate the effects of the color manipulation at Time 2, because the color should activate the beliefs only in those individuals who hold the beliefs. However, this approach is only possible if there are meaningful differences in beliefs between individuals. Even more challenging is the study of the third pathway, the effects of color on mental simulation. Physiological measures such as EEG, salivation measurements, or brain imaging can be used to assess desire for a food [60]. However, assessing concrete simulations, such as the perceived weight of a food, is difficult with such methods. Therefore, researchers could ask for such variables using self-report measures. An elegant but indirect way to test for simulation effects is to use an experimental design in which the simulation is made difficult in one experimental condition but not in another [61]. For example, researchers could disrupt the simulation of the perceived weight of a product in one condition by asking participants to hold a heavy weight, but not in another condition. If a color influences health judgments via the simulation of the perceived weight of the product, such a manipulation should moderate the effect with a reduced effect of the color in the condition where participants hold a heavy weight. 

Most of the studies reviewed on the influence of packaging color on health perceptions used self-report methods, such as ratings in questionnaires or comments in a focus group. These methods provide important indicators of the effects of color on a reflective level of product evaluation. However, self-report measures are not ideal for capturing implicit effects of color that are likely to influence spontaneous choices. Therefore, measures that tap into such immediate responses could complete the understanding of color effects. For example, the research by Mai et al. [21] and Tijssen et al. [23] mentioned in this review used the Implicit Association Test (IAT) [62]. The IAT is a measure that captures associations between categories that are likely to predict consumer choice when consumers focus on their affective responses and are less driven by thoughts about risk [63,64,65]. It has been used in food research as a measure that is less prone to social desirability bias, e.g., [66]. In addition, eye-tracking or physiological methods such as EEG could add to our understanding of color effects to measure users’ responses to color. In contrast to self-report measures and categorization tasks such as the IAT, these measures do not directly measure health judgments or associations with healthiness, but they could be important in illustrating the responses to color that accompany these judgments or associations. For example, eye-tracking could help us to understand whether certain colors influence attention to health information [67,68,69]. Furthermore, pupil diameter and the number of fixations could help us to understand affective responses and information processing evoked by color stimuli [69]. Physiological measures such as EEG could provide another measure to study such responses. Exposure to different color stimuli produces different neural activity in the brain, which can be measured by analyzing EEG signals [70,71].

In summary, the interplay between packaging color and consumers’ health perceptions of products cannot be underestimated. As consumers are constantly making health judgments in their daily life, often without the time or ability to objectively assess health, understanding the mechanisms by which they construct their judgments becomes important. This review and the introduction of cognitive ecological color theory provides a starting point for understanding how color, as an intrinsic component of product packaging, influences healthiness judgments across contexts, product types, and cultures. Future research is needed to test the implications of the proposed theoretical approach.

## Data Availability

No new data were created for this paper.
